# Quantitative Analysis of Ubiquitinated Proteins in Human Pituitary and Pituitary Adenoma Tissues

**DOI:** 10.3389/fendo.2019.00328

**Published:** 2019-05-22

**Authors:** Shehua Qian, Xiaohan Zhan, Miaolong Lu, Na Li, Ying Long, Xuejun Li, Dominic M. Desiderio, Xianquan Zhan

**Affiliations:** ^1^Key Laboratory of Cancer Proteomics of Chinese Ministry of Health, Xiangya Hospital, Central South University, Changsha, China; ^2^Hunan Engineering Laboratory for Structural Biology and Drug Design, Xiangya Hospital, Central South University, Changsha, China; ^3^State Local Joint Engineering Laboratory for Anticancer Drugs, Xiangya Hospital, Central South University, Changsha, China; ^4^Department of Neurosurgery, Xiangya Hospital, Central South University, Changsha, China; ^5^The Charles B. Stout Neuroscience Mass Spectrometry Laboratory, Department of Neurology, College of Medicine, University of Tennessee Health Science Center, Memphis, TN, United States; ^6^National Clinical Research Center for Geriatric Disorders, Xiangya Hospital, Central South University, Changsha, China

**Keywords:** pituitary adenoma, ubiquitination, quantitative proteomics, mass spectrometry, bioinformatics

## Abstract

Protein ubiquitination is an important post-translational modification that is associated with multiple diseases, including pituitary adenomas (PAs). Protein ubiquitination profiling in human pituitary and PAs remains unknown. Here, we performed the first ubiquitination analysis with an anti-ubiquitin antibody (specific to K-ε-GG)-based label-free quantitative proteomics method and bioinformatics to investigate protein ubiquitination profiling between PA and control tissues. A total of 158 ubiquitinated sites and 142 ubiquitinated peptides in 108 proteins were identified, and five ubiquitination motifs were found. KEGG pathway network analysis of 108 ubiquitinated proteins identified four statistically significant signaling pathways, including PI3K-AKT signaling pathway, hippo signaling pathway, ribosome, and nucleotide excision repair. R software Gene Ontology (GO) analysis of 108 ubiquitinated proteins revealed that protein ubiquitination was involved in multiple biological processes, cellular components, and molecule functions. The randomly selected ubiquitinated 14-3-3 zeta/delta protein was further analyzed with Western blot, and it was found that upregulated 14-3-3 zeta/delta protein in nonfunctional PAs might be derived from the significantly decreased level of its ubiquitination compared to control pituitaries, which indicated a contribution of 14-3-3 zeta/delta protein to pituitary tumorigenesis. These findings provided the first ubiquitinated proteomic profiling and ubiquitination-involved signaling pathway networks in human PAs. This study offers new scientific evidence and basic data to elucidate the biological functions of ubiquitination in PAs, insights into its novel molecular mechanisms of pituitary tumorigenesis, and discovery of novel biomarkers and therapeutic targets for effective treatment of PAs.

## Introduction

Pituitary adenomas (PAs) are a common type of intracranial tumor (1) that accounts for about 10% of intracranial tumors (2). Clinical manifestations include abnormalities of hormone secretion, pituitary apoplexy, compressing syndromes of tumor that surrounds the pituitary gland, and other anterior pituitary dysfunctions. PAs are divided into functional PAs (FPAs) and nonfunctional PAs (NFPAs) according to their hormone-secreting functions (3). FPAs display hormone hypersecretion. Because an FPA has its secondary symptoms and signs of excessive secretion of tumor hormones, it is easily diagnosed and treated at an earlier stage. NFPAs do not have any hormone hypersecretion and are more difficult to diagnosis (4). An NFPA usually has a larger volume at the time of diagnosis, and is often characterized by hypophysis dysfunction, visual field defect, and headache (5). Although a PA is commonly a benign tumor (6), secondary symptoms are caused by a large number of hormones produced by FPA and compression of surrounding tissues—causing vision loss, headaches, and so on by NFPA (7). Therefore, it is necessary to in-depth understand its molecular mechanisms of PAs and to discover novel biomarkers and therapeutic targets for effective treatment of PAs.

Protein ubiquitination is an important post-translational modification (PTM), which plays important roles in maintenance of the balance between protein synthesis and degradation, and in cell signaling, and associates with multiples diseases, including cancers (8, 9). For example, Luo et al. (10) found that TRAF6 regulates melanoma invasion and metastasis through ubiquitination of Basigin. Ubiquitin is a highly conserved small protein that consists of 76 amino acids, and is a heat-stable protein. In structure, ubiquitin is a polypeptide of 8.5 kDa (9, 11, 12). The full length of the ubiquitin molecule contains seven lysine sites (K6, K11, K27, K29, K33, K48, and K63), a methionine site at the N-terminus, and one glycine site at the C-terminus (13, 14). Ubiquitin can be covalently bound to the target protein under catalysis of a series of enzymes. This process is called ubiquitination. Ubiquitination is a process that modulates the PTMs of multiple cells and modifies protein function, stability, and localization (15–19). The ubiquitination process involves the synergy of three enzymes: ubiquitin-activating enzyme E1, ubiquitin-coupled enzyme E2, and ubiquitin ligase E3 (20–22). First, E1 utilizes the energy provided by ATP to form a high-energy thioester bond between the carboxyl group on the lysine C-terminal Lys residue and the thiol group on its own cysteine residue to activate the ubiquitin molecule. The activated ubiquitin is re-bound to the Cys residue of E2 through the thioester bond. Finally, the activated ubiquitin is either directly linked to the protein substrate via E2, or the ubiquitin is transferred to the ubiquitin to form an amino isopeptide bond between the carboxyl terminal of ubiquitin and the amino group of the Lys residue of the target protein under the action of E3 (9, 11). In this series of enzymatic cascades, E3 plays the most-important role in the specific recognition of target proteins and the regulation of ubiquitination system activity (20, 23), because E3 ligase recognizes substrates through specific protein-protein interactions (21). E1, E2, and E3 can form several different ubiquitination substrates. Some substrate proteins are only monoubiquitinated, and some have multiple lysine residues. Under appropriate conditions, multiple sites are monoubiquitinated, and some proteins form polyubiquitin chains at a single lysine site.

NFPAs are more common than FPAs in the population of PA patients. However, it is difficult for NFPA patients to obtain an early accurate diagnosis. The treatment of PA patients is generally surgery, radiotherapy, and chemotherapy; however, it is often difficult to achieve a complete cure. It is necessary to investigate new molecular mechanisms of pituitary tumorigenesis and biomarkers for treatment of PAs. Protein ubiquitination is one of PTMs that contribute to the generation of proteoforms. It is well-known that an important function of protein ubiquitination is in the degradation of proteins to maintain the balance of synthesis and degradation of proteins in human body. Furthermore, studies found that the ubiquitin proteasome system changes in pituitary adenomas (24), and that ubiquitination is involved in pituitary tumorigenesis (25). Our previous study also identified ubiquitin-proteasome, and that its proteasome subunit alpha type 2 was nitrated in pituitary adenomas, which affected the function of the proteasome that is a multicatalytic proteinase complex in the cytoplasmic and nuclear regions and is involved in an intracellular, ATP/ubiquitin-dependent, nonlysosomal proteolytic pathway (26). In addition, in our previous series of studies on PA comparative proteomics, an interesting phenomenon is that the number of down-regulated proteins is much more than the number of up-regulated proteins in PAs (27–30), the mRNA expression of ubiquitin-conjugating enzymes E2 and E3 was significantly increased in NFPAs (28), the mRNA expression of ubiquitin specific protease 34 was significantly decreased in PAs (29), ubiquitin carboxyl-terminal hydrolase isozyme L1 was identified in NFPAs (27), and the protein ubiquitination pathway was changed in NFPAs (30). Therefore, it is hypothesized that ubiquitination plays important roles in this interesting phenomenon to discover the key protein ubquitinations for in-depth insights into molecular mechanisms of NFPAs, and to discover reliable biomarkers and effective therapeutic targets. It is important to investigate protein ubiquitinations in human NFPAs.

Anti-ubiquitin antibody-based label-free quantitative proteomics is an effective method to globally detect, identify, and quantify protein ubiquination in a given condition, such as tumors vs. controls (31–43). Briefly, the total proteins extracted from tumor and control tissues were digested with trypsin, respectively. The ubiquitinated tryptic peptides in the tryptic peptide mixture were isolated and enriched with an anti-ubiquitin antibody specific to a K-ε-GG group. Isolated ubiquitinated peptides were analyzed with liquid chromatography-tandem mass spectrometry (LC-MS/MS). The MS/MS data were used to search protein database to identify proteins and determine ubiquitinated sites, and the level of protein ubiquitination was determined with MaxQuant algorithms. MaxQuant is the leading qualitative and quantitative algorithm for label-free quantitative proteomics.

Trypsin digestion, anti-ubiquitin antibody-based enrichment, and label-free quantitative proteomics are addressed here. The ubiquitin molecule is made up of 76 amino acids, and the C-terminal glycine is conjugated via its carboxy group to the amino group of a lysine side-chain or to the N-terminus. Trypsin digestion of ubiquitinated proteins cleaves off all but the two C-terminal glycine residues of ubiquitin from the modified protein. These two C-terminal glycine (GG) residues remain linked to the ε-amino group of the modified lysine residue in the tryptic peptide derived from digestion of the substrate protein. The presence of the GG on the side chain of that lysine prevents cleavage by trypsin at that site, to result in an internal modified lysine residue in a formerly ubiquitinated peptide. The K-ε-GG group is recognized and enriched with an antibody specific to K-ε-GG (37). For the ubiquitinated K (Ub-K) residue at the C-terminus, N-terminus, or the middle in a peptide, it mainly results from steric bulk hindrance, which does not affect the MS identification (42). The distinct mass shift (114.04 Da) caused by the GG remnant enables identification and precise localization of ubiquitylation sites based on peptide fragments (41). The identified ubiquitinated peptides were quantified with MaxQuant algorithms (43).

This study used anti-ubiquitin antibody-based label-free quantitative proteomics to identify ubiquitinated proteins and sites, and to quantify the level of ubiquitination. Pathway network analysis was used to investigate any molecular network alteration that protein ubiquitination is involved in. Selected ubiquitinated proteins were further analyzed to reveal the roles of ubiquitinated proteins in PAs. These findings will help to elucidate the molecular mechanisms, and discover biomarkers and therapeutic targets for PAs.

## Materials and Methods

### Tissue Samples

Eight PA tissues were obtained from the Department of Neurosurgery of Xiangya Hospital, China, as approved by the Xiangya Hospital Medical Ethics Committee of Central South University. Post-mortem control pituitary tissues were obtained from the Memphis Regional Medical Center (*n* = 5), as approved by the University of Tennessee Health Science Center Internal Review Board. Written informed consent was obtained from each patient or the family of each control pituitary subject (post-mortem tissues) after full explanation of the purpose and nature of all experimental procedures. The detailed information of PA and control pituitary tissue samples are collected in Table 1. Quantitative ubiquitination proteomics was performed between the four mixed NFPA samples and the four mixed control samples. Western blot experiments were performed between the six mixed NFPA samples and the three mixed control samples.

**Table 1:** Clinical characteristic of NFPA and control tissue samples.

**Group**	**Sex**	**Age**	**Clinical information**	**Immunohistochemistry**	**Experiments**
Control	Female	40	White, Multiple toxic compounds. Blood: HepB (+), HepC (+), HIV(–).	DNT	Proteomics; Western blot
	Male	45	White, Drowning. Blood alcohol = 3.1 g/L; no other drugs detected. Blood: HepB (+), HepC (+), HIV (–).	DNT	Western blot
	Male	36	White, Multiple toxic materials. Blood alcohol = 0.5 g/L. Blood: HepB (+), HepC (–), HIV (–).	DNT	Proteomics; Western blot
	Female	34	Black, Gunshot wound to chest. Blood alcohol = 0.3 g/L; no drugs. Blood: HepB (+), HepC (–), HIV (–).	DNT	Proteomics
	Female		White, 15 h gunshot wound to head. No drugs or alcohol. Blood: HepB (–), HepC (–), HIV (–).	DNT	Proteomics
NFPA	Female	43	NFPA in sellar region. Sellar floor bone thinning, enriched blood supply in tumor, and tumor size: 4 × 3 × 3 cm^3^	ACTH (–), hGH (–), PRL (–), FSH (+), LH (–), TSH (–)	Proteomics
	Male	53	NFPA in sellar region. Sellar floor bone thinning, and tumor size: 3 × 3 × 2.5 cm^3^	ACTH (–), hGH (–), PRL (–), FSH (–), LH (–), TSH (–)	Proteomics
	Female	43	NFPA in sellar region. Adhesion of surrounding tissues, and tumor size: 4.5 × 4 × 6 cm^3^	ACTH (–), hGH (–), PRL (–), FSH (+), LH (–), TSH (–)	Proteomics; Western blot
	Male	58	NFPA in sellar region. Sellar floor bone destruction, enriched blood supply in tumor, and tumor size: 4.5 × 3 × 3 cm^3^	ACTH (–), hGH (–), PRL (–), FSH (–), LH (–), TSH (–)	Proteomics; Western blot
	Male	40	NFPA in sellar region. Recurrent tumor, old blooding in tumor, and tumor size 2 × 2 × 1.8 cm^3^.	ACTH(–), hGH(–), PRL(–), FSH(+), LH(–), TSH(–)	Western blot
	Male	59	NFPA in sellar region. Sellar floor bone thinning, and enriched blood supply in tumor, and tumor size 2.1 × 1.8 × 2 cm^3^.	ACTH(–), hGH(–), PRL(–), FSH(+), LH(–), TSH(–)	Western blot
	Male	49	NFPA in sellar region. Sellar floor bone thinning, old blooding in tumor, and tumor size 2 × 4 × 3 cm^3^.	ACTH(–), hGH(–), PRL(–), FSH(–), LH(–), TSH(–)	Western blot
	Female	53	NFPA in sellar region. Sellar floor bone thinning, enriched blood supply, and tumor size 3 × 3.5 × 2.5 cm^3^.	ACTH(–), hGH(–), PRL(–), FSH(–), LH(–), TSH(–)	Western blot

*DNT, do not test*.

### Cleavage and Quantification of Proteins

A volume (1 ml) of urea pyrolysis solution [20 mM 2-hydroxyethyl (HEPES), 9 M urea, 2.5 mM sodium pyrophosphate, 1 mM sodium orthovanadate, and 1 mM β-glycerophosphate, pH 8.0] was added to each tissue sample (100 mg) for an ice-bath ultrasonic treatment (100 W, 10 s, interval 10 s, 10 times). The solution was centrifuged (18,000 × g, 30 min, 4°C). The supernatant was the protein extraction, and its protein content was determined with a Bradford Protein Quantification Kit (YEASEN, Cat# 20202ES76).

### Enzymatic Hydrolysis of Proteins

Four NFPA protein samples (1.5 mg/each sample) were equally mixed as tumor protein sample (6 mg), and four control protein samples (1.5 mg/each sample) were equally mixed as control protein sample (6 mg). Each mixed sample (tumor; control) was equally divided into three parts (2 mg/part) for proteomics experiment. A volume of 1 M dithiothreitol (DTT) was added to each sample part (tumor: *n* = 3; control: *n* = 3) to produce a final concentration of 10 mM; the mixture was incubated (37°C for 2.5 h), and cooled to room temperature. A volume of 1 M iodoacetamide was added to the mixture to achieve a final concentration of 50 mM, and the mixture was incubated in the dark for 30 min. Five volumes of water were added to dilute the urea concentration to 1.5 M. Trypsin (2 μg/μL) was added at 1:50 (v:v) and the mixture was digested (37°C for 18 h). The tryptic peptide mixture was desalted and lyophilized with an SPE C18 column (Waters WAT051910).

### Enrichment of Ubiquitinated Peptides

Each lyophilized tryptic peptide sample (tumor; control) was reconstituted in 1.4 mL of pre-cooled immunoaffinity purification (IAP) buffer. The pretreated anti-K-ε-GG antibody beads [PTMScan ubiquitin remnant motif (K-ε-GG) kit, Cell Signal Technology] were added. The mixture was incubated (1.5 h at 4°C) and centrifuged (2,000 × g, 30 s, 4°C). The supernatant was discarded (44). The pretreated anti-K-ε-GG antibody beads with peptides were washed three times with 1 mL of pre-cooled IAP buffer, and washed three times with 1 mL of pre-chilled water. After washing the anti-K-ε-GG antibody beads with peptides, 40 μL of 0.15% trifluoroacetate (TFA) was added. The mixture was incubated at room temperature for 10 min and centrifuged (30 s at 2,000 × g). The above incubation with 0.15% TFA and centrifugation steps (2,000 × g, 30 s) was performed three times. The supernatant that contained ubiquitinated peptides was desalted with C18 STAGE Tips (45).

### LC-MS/MS Analysis of Enriched Ubiquitinated Peptides

The enriched ubiquitinated peptides from PA and control tissues were analyzed with LC-MS/MS. Peptides of each sample were separated with a high performance liquid chromatography (HPLC) system EASY-nLC1000 at nanoliter flow rate. The solution A was 0.1% formic acid and 2% acetonitrile aqueous solution. The solution B was 0.1% formic acid and 84% acetonitrile aqueous solution. The chromatographic column was equilibrated with 100% solution A. The enriched ubiquitinated peptide sample was loaded with an autosampler onto a sample-spindle Thermo Scientific EASY column (2 cm^*^100 μm 5 μm-C18), and peptides were separated with an analytical column of 75 μm × 250 mm 3 μm-C18 at a flow rate of 250 nL/min. The HPLC liquid-phase gradients were as follows: solution B linear gradient from 0 to 55% during 0–220 min, solution B linear gradient from 55 to 100% during 220–228 min, and solution B maintained at 100% during 228–240 min. The enriched peptide products were separated with capillary HPLC according to the HPLC liquid-phase gradients within 240 min, and analyzed with a Q-Exactive mass spectrometer (Thermo Finnigan). The mass spectrometry (MS) detection was in the positive-ion mode. The scan range of the precursor ion was *m/z* 350–1800. The most-intense 20 ions in each MS spectrum were selected for higher-energy collisional dissociation (HCD) fragmentation for MS/MS analysis. The MS resolution was 70,000 at *m/z* 200, and the resolution of MS/MS was 17,500 at *m/z* 200. The enriched ubiquitinated peptide products from PAs or controls were analyzed three times with LC-MS/MS.

### Label-Free Analysis With MaxQuant

Six LC-MS/MS original files (tumor: *n* = 3; Control: *n* = 3) were imported into MaxQuant software (version 1.3.0.5) for database review, protein identification, ubiquitination-site determination, and quantification of ubiquitination level. The protein database was uniprot_human_154578_20160815.fasta (list of 154,578 entries, downloaded on 15 August 2016). The main search parameters were: main search ppm was 6, missed cleavage was 4, MS/MS tolerance ppm was 20, de-isotopic was TRUE, enzyme was trypsin, database was uniprot_human_154578_20160815.fasta, fixed modification was carbamidomethyl (C), variable modification was oxidation (M), acetyl (protein N-term), and GlyGly (K), decoy database pattern was reverse, iBAQ was TRUE, match between analyses was 2 min, peptide false discovery rate (FDR) was 0.01, and protein FDR was 0.01. Thus, the protein was characterized, ubiquitination site was determined with amino acid sequence analysis, and its ubiquitination level was quantified with MaxQuant algorithms.

### Statistical and Bioinformatics Analysis

The checksum files obtained by MaxQuant were analyzed with Perseus software (version 1.3.0.4). The DAVID database was used to perform KEGG signaling pathway-enrichment analysis of the ubiquitinated proteins. Gene ontology (GO) was used to annotate proteome with R software, and these ubiquitinated proteins were classified with GO annotation based on three categories: cellular components (CC), biological processes (BP), and molecular functions (MF). Motif-X software (http://motif-x.med.harvard.edu/motif-x.html) was used to analyze the model of ubiquitinated peptide sequences in specific positions of ubiquityl-31-mers (15 amino acid upstream and 15 amino acid downstream at the ubiquitination site) in all protein sequences. The International Protein Index (IPI) human proteome was used as the background database; setting parameters were width = 15, occurrences = 20, significance = 0.005; other parameters were set to default values.

### Western Blot Analysis of 14-3-3 Zeta/Delta Protein

Six NFPA protein samples were equally mixed as the tumor protein sample, and three control protein samples were equally mixed as the control protein sample (Table 1); the equal-load amount (tumor: 22 μg; control: 22 μg) of mixed samples were used for Western blot experiment. Based on the enriched signaling pathways, which include the PI3K-AKT signaling pathway and the Hippo signaling pathway, protein 14-3-3 zeta/delta was the key molecule, and was ubiquitinated in theses pathways; therefore, protein 14-3-3 zeta/delta was selected for Western blot (WB) analysis, and detailed experimental steps for protein extraction was described previously (46). The anti-protein 14-3-3 zeta/delta antibody (Cusabio, China) was diluted with Tris-buffered saline tween (TBST) (v1: v2 = 1:1000). The secondary antibody (Signalway Antibody, U.S.A.) was diluted with TBST (v1: v2 = 1:5000). The detailed experimental steps for WB were described previously (47–49). Briefly, proteins from PA and control samples were separated with 10% SDS-PAGE gel, transferred to a polyvinylidene fluoride (PVDF) membrane, incubated with anti-protein 14-3-3 zeta/delta antibody, incubated with secondary antibody, and visualized.

## Results

### Protein Ubiquitination in Control Pituitaries and Pituitary Adenomas

**Figure 1:**
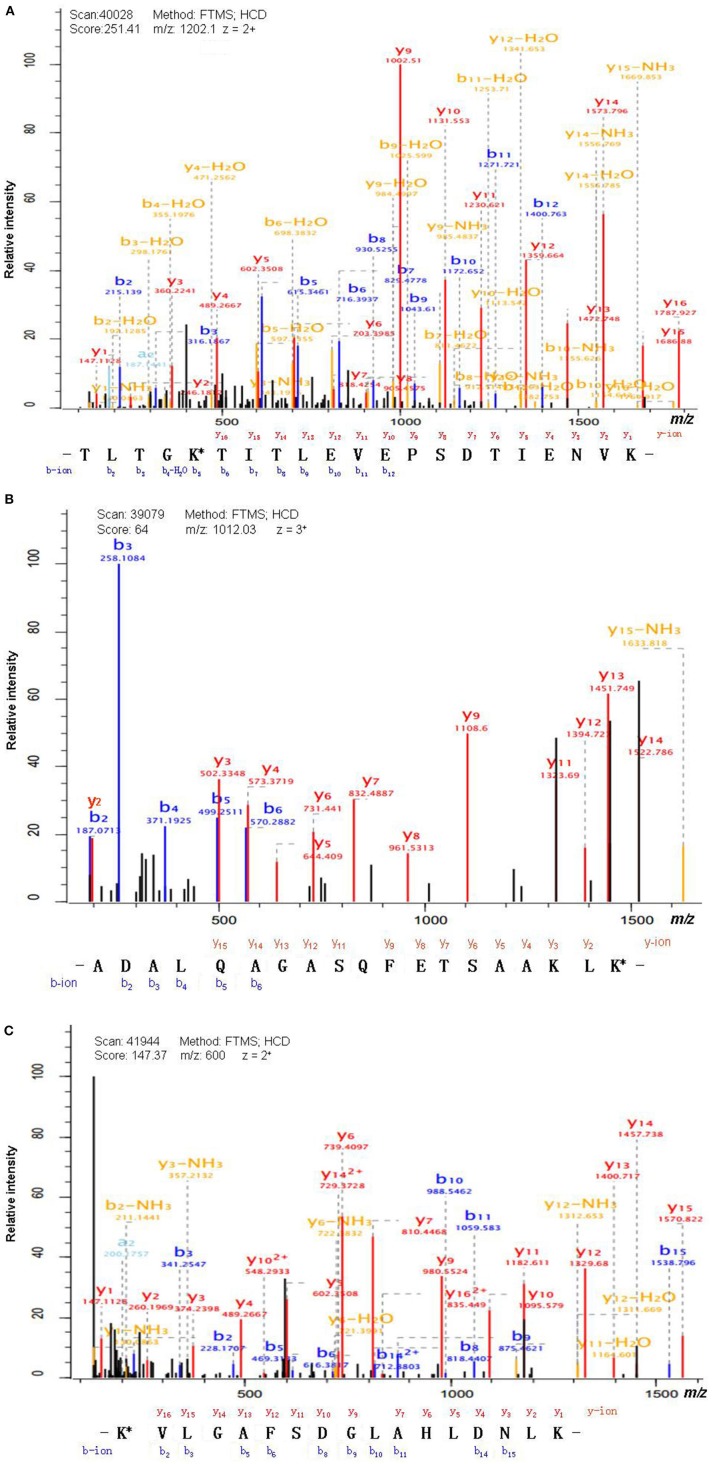
MS/MS spectrum of the tryptic peptide. **(A)** The tryptic peptide TLTGK*TITLEVEPSDTIENVK from epididymis luminal protein 112 (B2RDW1) or ubiquitin-40S ribosomal protein S27a (P62979). **(B)** The tryptic peptide ADALQAGASQFETSAAKLK* from uncharacterized protein (L7N2F9). **(C)** The tryptic peptide K*VLGAFSDGLAHLDNLK from hemoglobin subunit beta (P68871) or beta-globin (D9YZU5). The observed b- and y-ions were labeled in each MS/MS spectrum. K* = ubiquitinated lysine residue.

**Figure 2:**
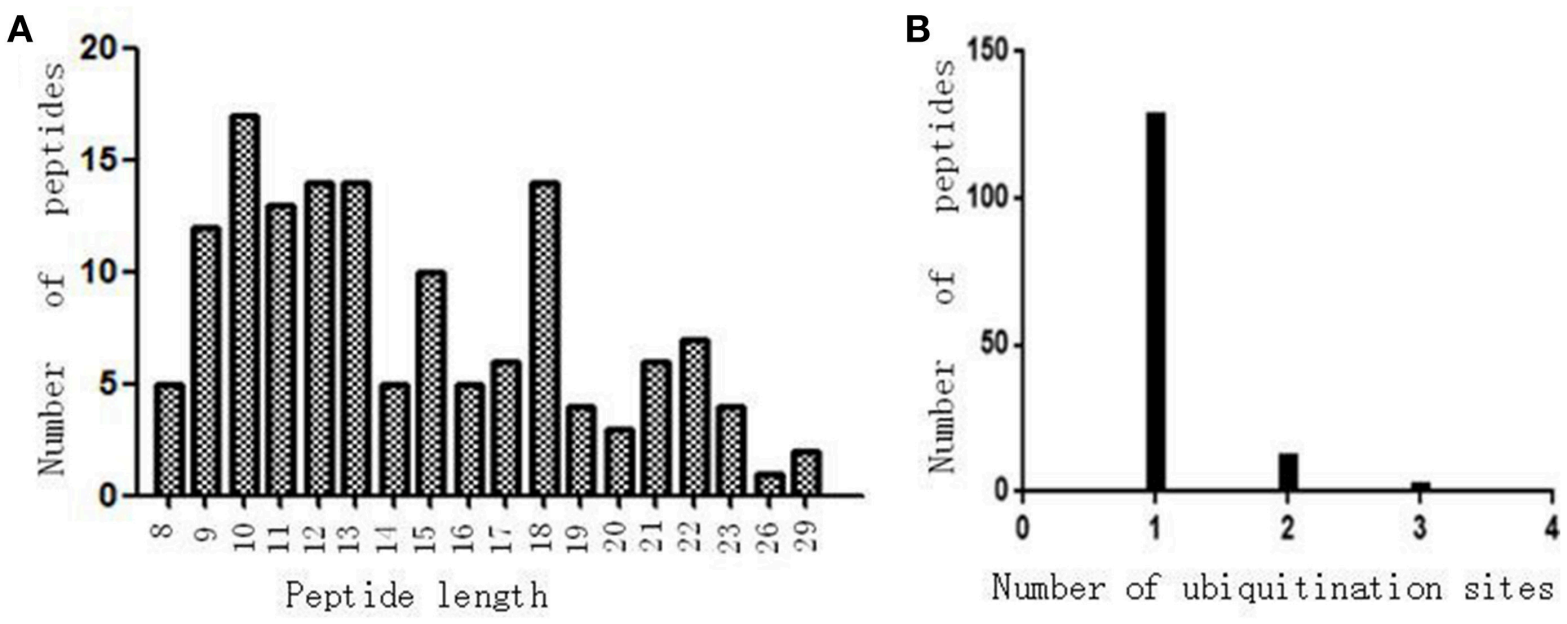
The ubiquitination profile in normal pituitaries and pituitary tumors. **(A)** Peptide length distribution of all ubiquitinated peptides. **(B)** Distribution of ubiquitinated peptides based on number of ubiquitination sites.

Antibody enrichment-based label-free quantitative proteomics identified 158 ubiquitinated sites and 142 ubiquitinated peptides from 108 proteins in PAs and control pituitaries (Table 2). A representative MS/MS spectrum was from ubiquitinated peptides ^7^TLTGK^*^TITLEVEPSDTIENVK^27^ ([M + 2H]^2+^, *m/z* = 1202.14; K^*^ = ubiquitinated lysine residue) of epididymis luminal protein 112 (B2RDW1) or ubiquitin-40S ribosomal protein S27a (P62979) (Figure 1A), with a high-quality MS/MS spectrum, excellent signal-to-noise (S/N) ratio, and extensive product-ion b-ion and y-ion series (b_2_, b_3_, b_4_-H_2_O, b_5_, b_6_, b_7_, b_8_, b_9_, b_10_, b_11_, and b_12_; y_1_, y_2_, y_3_, y_4_, y_5_, y_6_, y_7_, y_8_, y_9_, y_10_, y_11_, y_12_, y_13_, y_14_, y_15_, and y_16_). The ubiquitination site was localized to amino acid residue K${}_{11}^{*}$, and the ubiquitination level was significantly increased in PAs compared to controls (Table 2). Another representative MS/MS spectrum was from ubiquitinated peptide ^67^ADALQAGASQFETSAAKLK^*^^85^ of uncharacterized protein (L7N2F9) (Figure 1B), which localized the ubiquitination sites at K residue in the peptide C-terminal, with a high-quality MS/MS spectrum, excellent S/N ratio, and extensive product-ion b-ion and y-ion series (b_2_, b_3_, b_4_, b_5_, and b_6_; y_2_, y_3_, y_4_, y_5_, y_6_, y_7_, y_8_, y_9_, y_11_, y_12_, y_13_, y_14_, and y_15_). The ubiquitination site was localized to amino acid residue K${}_{85}^{*}$, and the protein was ubiquitinated in PAs but not in controls (Table 2). The third representative MS/MS spectrum was from ubiquitinated peptide ^67^K^*^VLGAFSDGLAHLDNLK^83^ of hemoglobin subunit beta (P68871) or beta-globin (D9YZU5) (Figure 1C), which localized the ubiquitination site at K residue in the peptide N-terminal, with a high-quality MS/MS spectrum, excellent S/N ratio, and extensive product-ion b-ion and y-ion series (b_2_, b_3_, b_5_, b_6_, b_8_, b_9_, b_10_, b_11_, b_14_, and b_15_; y_1_, y_2_, y_3_, y_4_, y_5_, y_6_, y_7_, y_9_, y_10_, y_11_, y_12_, y_13_, y_14_, y_15_, and y_16_). The ubiquitination site was localized to amino acid residue K${}_{67}^{*}$, and the ubiquitination level was significantly increased in PAs compared to controls (Table 2). With the same method, each ubiquitinated peptide and ubiquitination site was identified with MS/MS data, and quantified. Among the 142 ubiquitinated peptides that were identified, 45 ubiquitinated peptides were quantified in PA and control tissues, including 30 statistically significantly differentially ubiquitinated peptides in PAs compared to controls (*p* < 0.05). A total of 56 ubiquitinated peptides were quantified in PAs, but not in control pituitaries, and six ubiquitinated peptides were quantified in control pituitaries, but not in PAs. A total of 35 ubiquitinated peptides were identified but not quantified in PAs and controls. Moreover, most ubiquitinated peptides were 8–22 amino acids long (Figure 2A). Among 142 ubiquitinated peptides 90.1% (128/142) peptides contained only one ubiquitinated site, 8.5% peptides contained two ubiquitinated sites, 7.8% peptides contained three ubiquitinated sites, and 1.4% peptides contained over three ubiquitinated sites (Figure 2B).

**Table 2:** Ubiquitinated proteins identified in nonfunctional pituitary adenomas relative to controls.

**Accession No**.	**Gene name**	**Protein name**	**Modified peptides**	**Peptide length**	**Modified positions**	**Modified probabilities**	**Modified level (N)**	**Modified level (T)**	**Ratio (T/N)**	***t-*test *p-*value**
A0A0A6YY96	IREB2	Iron-responsive element-binding protein 2	GFQIAAEK*QK*	10	483;485	1; 1	3.59E+07	1.62E+08	4.52	4.55E-06
P16104	H2AFX	Histone H2AX	K*TSATVGPK	9	120	1	3.50E+07	1.43E+09	40.96	1.39E-05
P48200	IREB2	Iron-responsive element-binding protein 2	GFQIAAEK*QK*	10	483;485	1; 1	3.46E+07	1.63E+08	4.71	3.31E-05
B2RDW1	RPS30A	Epididymis luminal protein 112	TLTGK*TITLEVEPSDTIENVK	21	11	1	1.70E+08	1.26E+09	7.40	3.64E-05
			TLSDYNIQK*ESTLHLVLR	18	63	1	3.71E+07	1.64E+08	4.43	6.07E-03
			LIFAGK*QLEDGR	12	48	1	2.91E+09	7.32E+09	2.52	6.88E-03
			IQDK*EGIPPDQQR	13	33	1	2.68E+06	3.68E+07	13.74	9.55E-03
			TITLEVEPSDTIENVK*AK	18	27	0.956		1.95E+07		
			MQIFVK*TLTGK	11	6	0.923				
			TITLEVEPSDTIENVKAK*	18	29	0.594				
			VDENGK*ISR	9	113	1				
P62979	RPS27A	Ubiquitin-40S ribosomal protein S27a	TLTGK*TITLEVEPSDTIENVK	21	11	1	1.72E+08	1.27E+09	7.37	3.78E-05
			TLSDYNIQK*ESTLHLVLR	18	63	1	3.71E+07	1.96E+08	5.27	6.89E-04
P68871	HBB	Hemoglobin subunit beta	FFESFGDLSTPDAVMGNPK*VK*	21	60;62	0.5; 0.5	1.69E+06	2.01E+07	11.87	3.71E-04
			GTFATLSELHCDK*LHVDPENFR	22	96	1	1.89E+07	9.13E+07	4.83	1.23E-02
			K*VLGAFSDGLAHLDNLK	17	67	1	2.07E+06	1.02E+07	4.92	1.22E-01
L7N2F9		Uncharacterized protein	VNVDK*VLER	9	52	1	1.04E+07	1.23E+08	11.86	8.99E-04
			ADALQAGASQFETSAAK*LK	19	83	0.876		2.01E+07		
			ADALQAGASQFETSAAKLK*	19	85	0.539		4.61E+07		
			DQK*LSELDDR	10	59	1	6.10E+06	5.24E+07	8.60	2.47E-03
D9YZU5	HBB	Beta-globin	FFESFGDLSTPDAVMGNPK*VK*	21	60;62	0.5; 0.5	2.48E+06	2.02E+07	8.14	1.14E-03
			K*VLGAFSDGLAHLDNLK	17	67	1	1.79E+06	1.02E+07	5.69	4.65E-02
P62979	RPS27A	Ubiquitin-40S ribosomal protein S27a	IQDK*EGIPPDQQR	13	33	1	3.34E+06	5.10E+07	15.27	1.16E-03
			LIFAGK*QLEDGR	12	48	1	2.90E+09	7.33E+09	2.53	6.90E-03
F5H5D3	TUBA1C	Tubulin alpha-1C chain1	VGINYQPPTVVPGGDLAK*VQR	21	370	1	2.27E+07	3.78E+07	1.67	1.38E-03
			DVNAAIATIK*TK*	12	336;338	0.679;0.57		2.98E+06		
Q9HCC9	ZFYVE28	Lateral signaling target protein 2	DFCVK*FPEEIR	11	87	1	7.36E+06	2.31E+07	3.14	1.50E-03
A0A024R017	HIST1H2AC	Histone H2A	NDEELNK*LLGR	11	96	1		3.45E+06		
			VTIAQGGVLPNIQAVLLPK*K*	20	119;120	0.5; 0.5	6.31E+07	1.13E+08	1.80	1.52E-03
P69905	HBA1	Hemoglobin subunit alpha	AAWGK*VGAHAGEYGAEALER	20	17	1		7.09E+06		
			TYFPHFDLSHGSAQVK*	16	57	1	1.94E+06	2.06E+08	106.34	5.10E-03
P08670	VIM	Vimentin	K*LLEGEESR	9	402	1	1.70E+06	5.54E+06	3.26	5.62E-03
			EK*LQEEMLQR	10	188	1	1.40E+07	2.02E+07	1.44	2.44E-01
			QVDQLTNDK*AR	11	168	1	1.99E+06	2.97E+06	1.49	2.74E-01
			K*LLEGEESR	9	393	1		5.54E+06		
C8C504	HBB	Beta-globin	GTFATLSELHCDK*LHVDPENFR	22	96	1	2.04E+07	8.43E+07	4.14	5.79E-03
Q96T46	HBA2	Hemoglobin alpha 2	TYFPHFDLSHGSAQVK*	16	33	1	1.94E+06	2.03E+08	104.44	6.08E-03
Q9H582	ZNF644	Zinc finger protein 644	RSFLQQDVNK*	10	11	1	9.99E+06	4.56E+07	4.56	6.13E-03
Q9H3H9	TCEAL2	Transcription elongation factor A protein-like 2	QYK*EAIHDMNFSNEDMIR	18	154	1	3.49E+06	1.77E+07	5.07	6.49E-03
E9PL57	NEDD8-MDP1	Protein NEDD8-MDP1	TLTGK*EIEIDIEPTDKVER	19	11	1	3.50E+06	1.20E+07	3.43	6.76E-03
Q59EJ3		Heat shock 70kDa protein 1A variant	MVQEAEK*YK	9	592	0.891	9.34E+05	9.36E+06	10.02	1.11E-02
D6R956	UCHL1	Ubiquitin carboxyl-terminal hydrolase	CFEK*NEAIQAAHDAVAQEGQCR	22	135	1		5.86E+06		
			MQLK*PMEINPEMLNK	15	4	1	2.77E+06	8.80E+06	3.17	1.61E-02
P14735	IDE	Insulin-degrading enzyme	EVNAVDSEHEK*NVMNDAWR	19	192	1	2.05E+06	7.47E+06	3.65	1.94E-02
Q6ZUF2		cDNA FLJ43763 fis, clone TESTI2048603	FFSPPNMSVTHK*EAHERK	18	19	0.977	1.39E+08	2.50E+08	1.80	5.15E-02
P61088	UBE2N	Ubiquitin-conjugating enzyme E2 N	ICLDILKDK*	9	94	0.962	1.74E+07	2.27E+07	1.30	7.06E-02
D1MGQ2	HBA2	Alpha-2 globin chain	AAWGK*VGAHAGEYGAEALER	20	17	1		7.09E+06		
			TYFPHFDLSHGSAQVK*	16	57	1	2.22E+06	1.26E+08	56.71	1.21E-01
P02042	HBD	Hemoglobin subunit delta	GTFSQLSELHCDK*LHVDPENFR	22	96	1	1.57E+07	2.01E+07	1.28	1.43E-01
D3DQ48	TMEM59	Transmembrane protein 59, isoform CRA_e	TEDHEEAGPLPTK*VNLAHSEI	21	316	1	3.64E+06	1.31E+07	3.61	1.63E-01
K7EPI4	GFAP	Glial fibrillary acidic protein	GGK*STK*DGENHK*	12	10;13;19	1;1;1	2.32E+06	2.06E+07	8.87	2.34E-01
A0A024R5H9	PLEKHB1	Pleckstrin homology domain containing, family B (Evectins) member 1, isoform CRA_a	LHLCAETK*DDALAWK	15	113	0.999	6.23E+06	0.00E+00	0.00	1.39E-01
B7WPA5	PLEKHB2	Pleckstrin homology domain-containing family B member 2	QNIEDK*VHMPMDCINIR	17	46	1	1.30E+07	7.08E+06	0.54	3.41E-01
Q3BBV0	NBPF1	Neuroblastoma breakpoint family member 1	VGWALDMDEIEK*	12	950	1	8.76E+06	5.34E+06	0.61	4.18E-01
A4UGR9	XIRP2	Xin actin-binding repeat-containing protein 2	WLFETQPMESLYEK*	14	934	1	1.82E+07	2.61E+07	1.44	5.39E-01
B7ZMF0	CEP192	CEP192 protein	IVSPK*NSDLK	10	556	0.908	1.14E+07	1.96E+07	1.71	6.03E-01
A0N071	HBD	Delta globin	GTFSQLSELHCDK*LHVDPENFR	22	96	1	2.08E+07	2.20E+07	1.05	8.73E-01
A0A024QZP6	H2AFY2	Core histone macro-H2A	IHPELLAK*K*	9	116;117	0.705; 0.585				
A0A024R466	ITM2C	Integral membrane protein 2C, isoform CRA_a	ISFQPAVAGIK*GDK	14	14	0.855		1.33E+07		
A0A024R6B3	TMEM63C	Transmembrane protein 63C, isoform CRA_a	DIEDPELIIK*HFHEAYPGSVVTR	23	239	1		3.89E+06		
A0A024R7G8	RAD23A	RAD23 homolog A (*S. cerevisiae*), isoform CRA_a	LIYAGK*ILSDDVPIR	15	53	1		1.24E+07		
A0A024RDB0	UBE1L2	Ubiquitin-activating enzyme E1-like 2, isoform CRA_a	IDAHLNK*VCPTTETIYNDEFYTK	23	544	1		4.66E+06		
A0A087WZE4	SPTA1	Spectrin alpha chain, erythrocytic 1	VNILTDK*SYEDPTNIQGK	18	79	1		6.34E+06		
A0A0K0KMI2	POU5F1	POU class 5 homeobox 1 transcript variant OCT4B4	K*LGGQLGR	8	1	1		7.82E+06		
A0A0U1RRH7		Histone H2A	NDEELNK*LLGK	11	96	0.996				
A0A140VJZ1		Testicular tissue protein Li 218	LEK*IFQNAPTDPTQDFSTQVAK	22	360	1				
A0AVT1	UBA6	Ubiquitin-like modifier-activating enzyme 6	IDAHLNK*VCPTTETIYNDEFYTK	23	544	1		4.54E+06		
A4D2P6	GRID2IP	Delphilin	GK*MGTVSK*SR	10	570;576	1; 1				
A6NNT2	C16orf96	Uncharacterized protein C16orf96	SALAGK*ASR	9	819	1				
A6ZKI3	FAM127A	Protein FAM127A	K*ESPLLNDYR	10	86	1		1.64E+07		
A8K6M4		cDNA FLJ75725, highly similar to Homo sapiens vesicle transport through interaction with t-SNAREs homolog 1B (yeast) (VTI1B), mRNA	ASSAASSEHFEK*LHEIFR	18	13	1		9.09E+06		
B1A4G6	GH1	Growth hormone 1 isoform 1	EETQQK*SNLELLR	13	96	1	5.87E+06			
B4DPP6		cDNA FLJ54371, highly similar to Serum albumin	K*LVAASQAALGL	12	607	1				
			K*QTALVELVK	10	558	1				
B4DR52		Histone H2B	HAVSEGTK*AVTK	12	117	1		4.70E+07		
B4E1J8		cDNA FLJ56285, highly similar to ADP-ribosylation factor-like protein 8B	DLPNALDEK*QLIEK	14	193	1				
B9EGU6	RC3H1	Ring finger and CCCH-type zinc finger domains 1	KEIMAQLEERK*	11	769	0.999		7.15E+06		
C9JLQ8	AEBP1	Adipocyte enhancer-binding protein 1	MPPEKTK*DK*	9	7;9	0.985;1				
E7EX29	YWHAZ	14-3-3 protein zeta/delta	MDKNELVQKAK*	11	11	0.537	2.35E+06			
K7EPF9	APOC1	Apolipoprotein C-I	EFGNTLEDK*AR	11	47	1		7.55E+06		
M1VKI3	SDC4-ROS1_S4;R32	Tyrosine-protein kinase receptor	AGSGSQVPTEPKK*	13	105	0.554		1.53E+07		
P01241	GH1	Somatotropin	EETQQK*SNLELLR	13	96	1	4.23E+06			
P02671	FGA	Fibrinogen alpha chain	EK*VTSGSTTTTR	12	448	1		4.06E+06		
			TVIGPDGHK*EVTK	13	476	0.86				
P02768	ALB	Serum albumin	K*LVAASQAALGL	12	598	1				
			K*QTALVELVK	10	549	1				
P04908	HIST1H2AB	Histone H2A type 1-B/E	NDEELNK*LLGR	11	96	1		3.45E+06		
P09960	LTA4H	Leukotriene A-4 hydrolase	FSYK*SITTDDWK	12	418	1		4.10E+06		
P19021	PAM	Peptidyl-glycine alpha-amidating monooxygenase	AFGDSEHK*LETSSGR	15	901	1				
P23396	RPS3	40S ribosomal protein S3	KPLPDHVSIVEPK*DEILPTTPISEQK	26	214	1		2.84E+07		
P31946	YWHAB	14-3-3 protein beta/alpha	SELVQKAK*	8	13	0.672				
P35212	GJA4	Gap junction alpha-4 protein	ALPAK*DPQVER	11	119	1		2.26E+06		
P35251	RFC1	Replication factor C	KLVSETVK*	8	22	1				
P45974	USP5	Ubiquitin carboxyl-terminal hydrolase 5	LEK*IFQNAPTDPTQDFSTQVAK	22	360	1		9.83E+06		
P54725	RAD23A	UV excision repair protein RAD23 homolog A	LIYAGK*ILSDDVPIR	15	53	1		1.39E+07		
			LIYAGK*ILNDDTALK	15	51	0.993				
P54727	RAD23B	UV excision repair protein RAD23 homolog B	LIYAGK*ILNDDTALK	15	51	0.993				
P55072	VCP	Transitional endoplasmic reticulum ATPase	ASGADSK*GDDLSTAILK	17	8	1		7.91E+06		
P60709	ACTB	Actin, cytoplasmic 1	DSYVGDEAQSK*R	12	61	1		2.05E+06		
P60866	RPS20	40S ribosomal protein S20	DTGK*TPVEPEVAIHR	15	8	1		9.48E+06		
P61981	YWHAG	14-3-3 protein gamma	EQLVQK*AR	8	10	1				
P62979	RPS27A	Ubiquitin-40S ribosomal protein S27a	TITLEVEPSDTIENVK*AK	18	27	0.956		1.72E+07		
			MQIFVK*TLTGK	11	6	0.923				
			TITLEVEPSDTIENVKAK*	18	29	0.594				
			VDENGK*ISR	9	113	1				
P83916	CBX1	Chromobox protein homolog 1	KEESEK*PR	8	109	1				
Q0VDD8	DNAH14	Dynein heavy chain 14, axonemal	SLLSNVSQWDTFK*	13	2919	1				
Q13753	LAMC2	Laminin subunit gamma-2	ITSTFHQDVDGWK*	13	219	1		1.99E+07		
Q15149	PLEC	Plectin	SELELTLGK*LEQVR	14	1210	1		2.72E+06		
Q4LE39	ARID4B	AT-rich interactive domain-containing protein 4B	K*ENIK*PSLGSK*	11	462;466;472	1;1;1		2.05E+07		
Q59EQ3		Nudix-type motif 6 isoform a variant	LDAAAFQK*GLQGK*	13	84;89	1;1		5.22E+06		
Q5I0G2	PRL	Growth hormone A1	AVEIEEQTK*R	10	153	1		5.21E+06		
Q5JWF2	GNAS	Guanine nucleotide-binding protein G(s) subunit alpha isoforms XLas	IDVIK*QADYVPSDQDLLR	18	829	1				
Q5VXU3	CHIC1	Cysteine-rich hydrophobic domain-containing protein 1	SIQK*LIEWENNR	12	176	1				
Q92625	ANKS1A	Ankyrin repeat and SAM domain-containing protein 1A	GK*EQELLEAAR	11	3	1		8.07E+06		
Q93045	STMN2	Stathmin-2	DLSLEEIQKK*	10	87	0.569		2.05E+06		
			DLSLEEIQK*K	10	86	0.5				
Q93100	PHKB	Phosphorylase b kinase regulatory subunit beta	AYLQLGINEK*	10	546	1	2.91E+07			
Q96AP7	ESAM	Endothelial cell-selective adhesion molecule	ALEEPANDIK*EDAIAPR	17	286	1				
Q96EI5	TCEAL4	Transcription elongation factor A protein-like 4	EYK*EAIHDMNFSNEDMIR	18	142	1		8.59E+06		
Q96N64	PWWP2A	PWWP domain-containing protein 2A	TGLEK*MRSGK*	10	496;501	1; 1	1.06E+07			
Q9BT67	NDFIP1	NEDD4 family-interacting protein 1	TK*AEATIPLVPGR	13	83	1				
Q9BUL8	PDCD10	Programmed cell death protein 10	QILSK*IPDEINDR	13	116	1		2.54E+07		
Q9BWQ8	FAIM2	Protein lifeguard 2	APGTEGQQQVHGEK*K	15	25	0.822		2.50E+06		
Q9H3Z4	DNAJC5	DnaJ homolog subfamily C member 5	FK*EINNAHAILTDATK	16	58	1		4.81E+06		
Q9H598	SLC32A1	Vesicular inhibitory amino acid transporter	DQVGGGGEFGGHDK*PK	16	113	0.832		9.03E+06		
Q9NQX7	ITM2C	Integral membrane protein 2C	ISFQPAVAGIK*GDK	14	14	0.855		1.33E+07		
Q9NV96	TMEM30A	Cell cycle control protein 50A	DEVDGGPPCAPGGTAK*TR	18	24	1		3.72E+06		
Q9NX12		cDNA FLJ20496 fis, clone KAT08729	SGEEALIIPPDAVAVDCK*DPDDVVPVGQR	29	39	1		6.69E+06		
			VTFNSALAQK*EAK	13	13	0.733				
Q9P0M6	H2AFY2	Core histone macro-H2A.2	IHPELLAK*K*	9	116;117	0.705; 0.585				
Q9P1W3	TMEM63C	Calcium permeable stress-gated cation channel 1	DIEDPELIIK*HFHEAYPGSVVTR	23	239	1		5.57E+06		
Q9UBB4	ATXN10	Ataxin-10	ITSDEPLTK*DDIPVFLR	17	262	1		1.14E+07		
Q9UEU0	VTI1B	Vesicle transport through interaction with t-SNAREs homolog 1B	ASSAASSEHFEK*LHEIFR	18	13	1		9.35E+06		
Q9UF11	PLEKHB1	Pleckstrin homology domain-containing family B member 1	LHLCAETK*DDALAWK	15	113	0.999	6.23E+06			
Q9UKJ5	CHIC2	Cysteine-rich hydrophobic domain-containing protein 2	SIEK*LLEWENNR	12	117	1		4.92E+06		
Q9UQB3	CTNND2	Catenin delta-2	SPSIDSIQK*DPR	12	540	1		3.06E+06		
Q9Y287	ITM2B	Integral membrane protein 2B	SGEEALIIPPDAVAVDCK*DPDDVVPVGQR	29	39	1		6.69E+06		
			VTFNSALAQK*EAK	13	13	0.733				
S4R435	RPS10-NUDT3	Protein RPS10-NUDT3	SAVPPGADKK*	10	139	0.574		5.99E+07		
			SAVPPGADK*K	10	138	0.846		6.91E+07		
U6FSN9	Mprip-Ntrk1 fusion gene	Tyrosine-protein kinase receptor	GWLTK*QYEDGQWK	13	396	1				

Ratio (T/N), Ration of tumors (B) to controls(A). K^*^, Ubiquitinated lysine residue.

### Signaling Pathways Involved in Ubiquitinated Proteins

**Figure 3:**
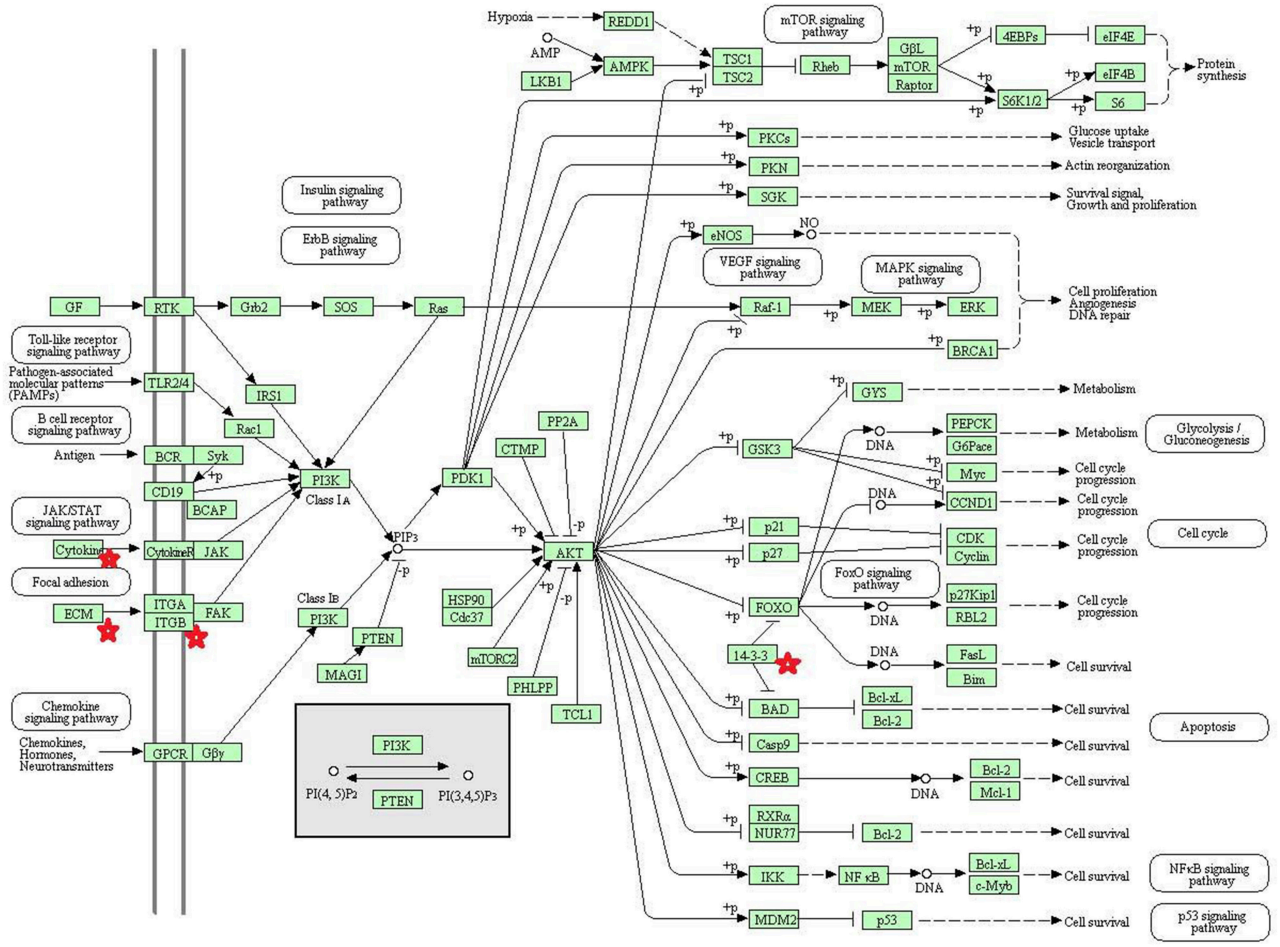
The PI3K-AKT signaling pathway which was achieved by DAVID pathway analysis. The ubiquitinated proteins are shown by red stars.

**Figure 4:**
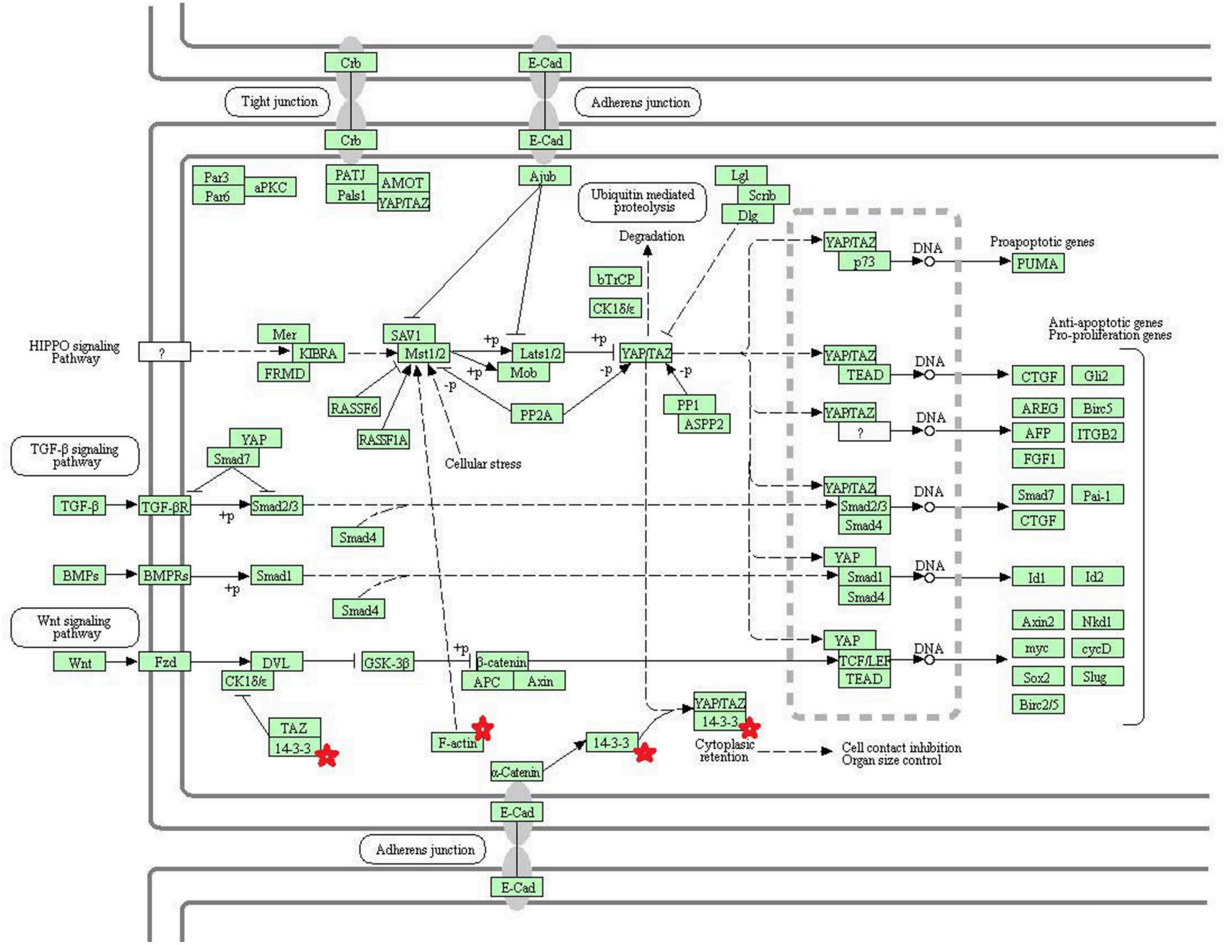
The Hippo signaling pathway which was achieved by DAVID pathway analysis. The ubiquitinated subunits are shown by red stars.

**Figure 5:**
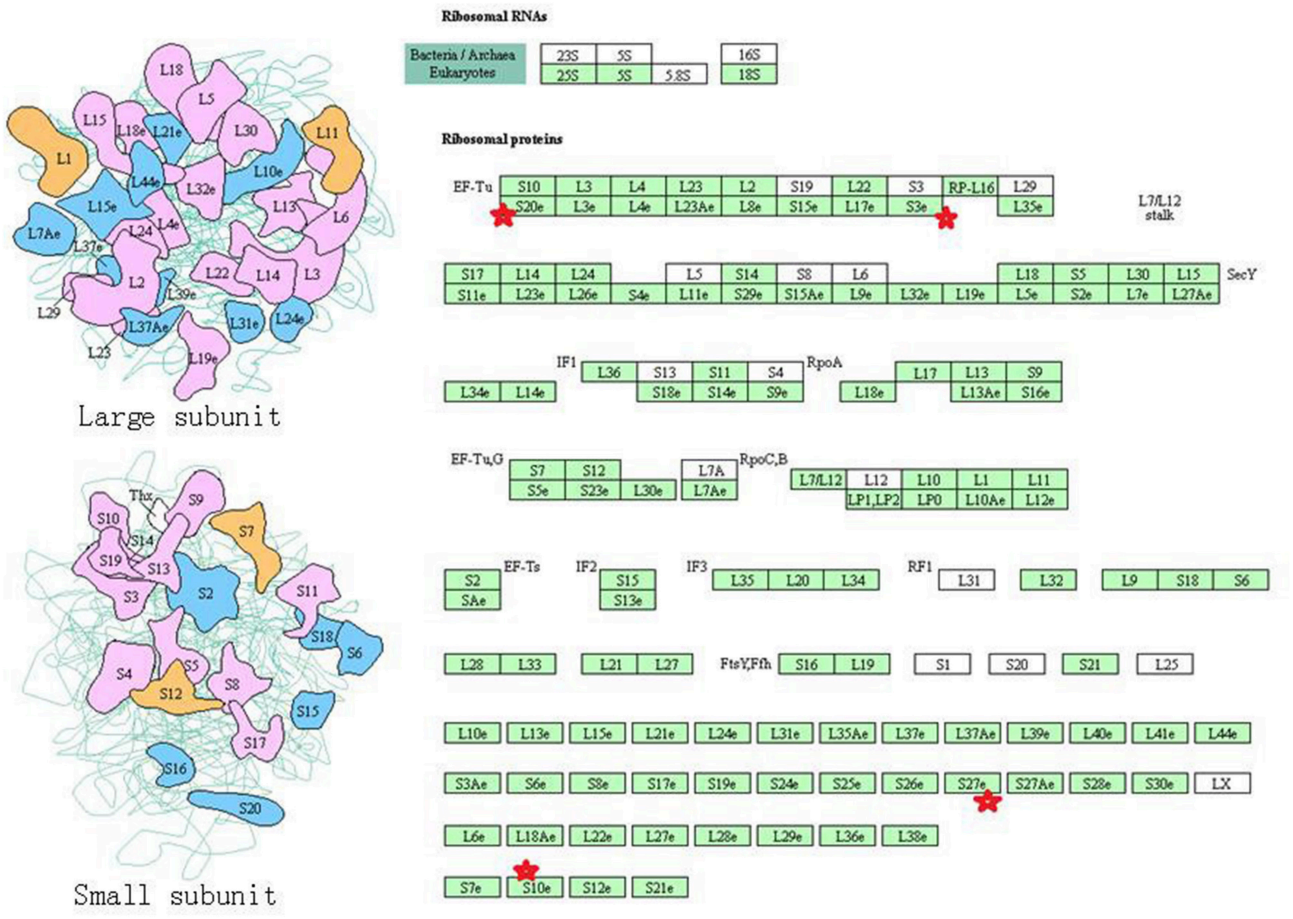
The ribosome signaling pathway which was achieved by DAVID pathway analysis. The ubiquitinated subunits are shown by red stars.

**Figure 6:**
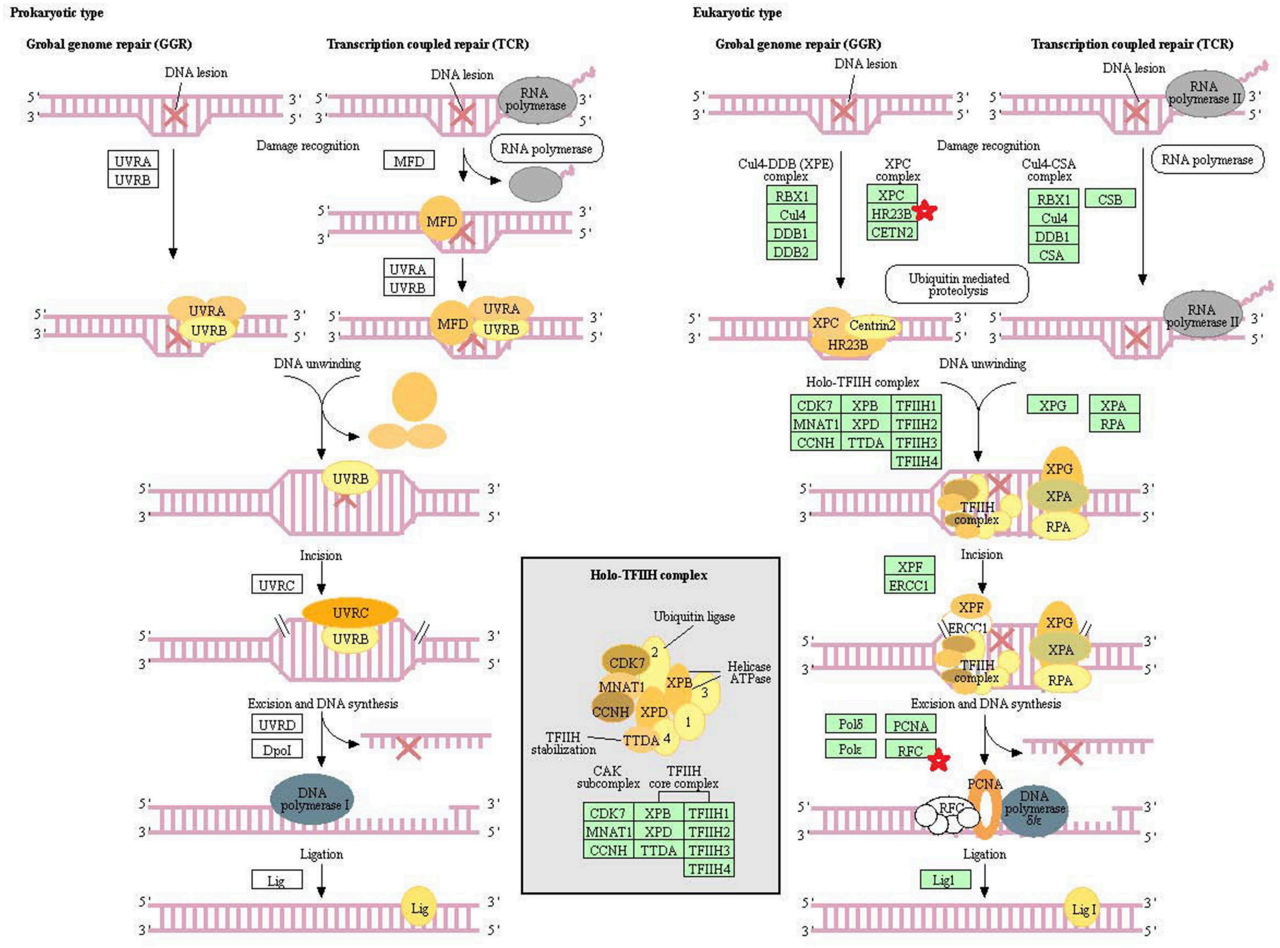
The nucleotide excision repair which was achieved by DAVID pathway analysis. The ubiquitinated subunits are shown by red stars.

Eight statistically significant KEGG signaling pathways (*p* < 0.05) were identified with DAVID KEGG pathway-enrichment analysis from 108 ubiquitinated proteins, including PI3K-AKT signaling pathway, Hippo signaling pathway, ribosome, nucleotide excision repair, alcoholism, systemic lupus erythematosus, African trypanosomiasis, and malaria. Among them, PI3K-AKT signaling pathway (Figure 3) is activated by many types of cellular stimulation and toxic damage, and regulates essential cellular functions such as transcription, translation, proliferation, survival, and growth. Hippo signaling pathway (Figure 4) is an evolutionarily conserved signaling pathway that controls the size of organs from flies to humans. For human and mouse, Hippo signaling pathway consists of MST1 and MST2 kinases, their cofactors Salvador, LATS1, and LATS2. In response to high cell density, activated LATS1/2 phosphorylates the transcriptional coactivators YAP and TAZ to promote their cytoplasmic localization, leads to apoptosis, and limits organ-size overgrowth. When the Hippo signaling pathway is inactivated at low cell densities, YAP/TAZ translocate into the nucleus to bind to the transcriptional factor enhancer (TEAD/TEF) family to promote cell growth and proliferation. YAP/TAZ also interact with other transcriptional factors or signaling molecules to allow Hippo pathway-mediated processes interact with other key signaling cascade processes, such as TGF-β and Wnt signaling pathways. Because the function of ribosomes is to translate the genetic code (nucleotide sequence) on the mRNA into the amino acid sequence on the polypeptide chain, the ribosome is closely related to protein synthesis. Ribosome signaling pathway (Figure 5) was enriched, and indicated that certain ubiquitinated proteins are closely related to this signaling pathway to thus affect protein synthesis. Nucleotide Excision repair (NER) (Figure 6) is a mechanism to recognize and repair large amounts of DNA damage caused by compounds, environmental carcinogens, and ultraviolet radiation. Protein ubiquitination might be involved in the nucleotide excision repair process to affect protein synthesis and the corresponding biological functions in PAs. Therefore, protein ubiquitination participated in multiple signaling pathway systems and biological processes in human PAs.

### Functional Characteristics of Ubiquitinated Proteins

In order to further understand the biological function of the ubiquitinated proteins in the development of PAs, GO enrichment analysis of identified ubiquitinated proteins revealed multiple CCs, BPs, and MFs. For CC analysis (Table 3), 33 ubiquitinated proteins were assigned to different CCs. A large number of ubiquitinated proteins were located on ribosome and vesicle. It is well-known that ribosomes are complexes composed of rRNA and proteins, and are important sites for protein synthesis. In addition, vesicles and ribosomal subunits also play an important role in protein synthesis. Ubiquitinated proteins can be degraded by the proteasome pathway (8). When the protein on ribosome or vesicle is usually ubiquitinated, the protein might degrade and affect the synthesis and secretion of other proteins, affect the normal physiological function of the body, and lead to PAs. For BP analysis (Table 4), most ubiquitinated proteins were associated with some important biological processes such as cellular responses to certain substances, self-regulation of cells, DNA repair, etc. Abnormal DNA repair was involved in the occurrence and development of tumors (50). When the proteins involved in DNA repair were ubiquitinated, abnormal DNA repair might occur and lead to PAs. In PA patients, the primary treatment was surgery; however, prolactinomas were usually treated with dopamine agonists (51, 52). The ubiquitinated proteins were associated with drug transport, which might make it difficult for drugs in PA patients to get to the target and thus allow development of tumors. For MF analysis (Table 5), 31 ubiquitinated proteins were significantly enriched in different MFs. The molecular functions of enriched ubiquitinated proteins were mainly combined with other substances, such as oxygen, organic acids, cofactors, etc. An important tumor marker was the infinite proliferation of tumor cells and angiogenesis (53). The proliferation of cells and the production of new blood vessels were inseparable from oxygen and nutrients. Ubiquitinated proteins could bind to oxygen, which might affect the transport of oxygen and nutrients, to thus affect the occurrence and development of PAs.

**Table 3:** Statistically significant GO cellular components (CC) derived from ubiquitinated proteins in human pituitary adenomas.

**ID**	**Cellular components**	**GeneRatio**	**BgRatio**	***P*-value**	***P*.adjust**	***Q*-value**	**GeneID**	**Count**
GO:0022627	Cytosolic small ribosomal subunit	4/33	45/19659	9.34E-07	1.33E-04	8.26E-05	RPS3/RPS20/HBA1/HBA2	4
GO:0015935	Small ribosomal subunit	4/33	73/19659	6.60E-06	4.68E-04	2.92E-04	RPS3/RPS20/HBA1/HBA2	4
GO:0022626	Cytosolic ribosome	4/33	115/19659	3.99E-05	1.89E-03	1.18E-03	RPS3/RPS20/HBA1/HBA2	4
GO:0044445	Cytosolic part	5/33	250/19659	5.67E-05	2.01E-03	1.25E-03	IDE/RPS3/RPS20/HBA1/HBA2	5
GO:0072562	Blood microparticle	4/33	147/19659	1.04E-04	2.95E-03	1.83E-03	FGA/ALB/HBA1/HBA2	4
GO:0031838	Haptoglobin-hemoglobin complex	2/33	11/19659	1.49E-04	3.17E-03	1.97E-03	HBA1/HBA2	2
GO:0005833	Hemoglobin complex	2/33	12/19659	1.78E-04	3.17E-03	1.97E-03	HBA1/HBA2	2
GO:0060198	Clathrin-sculpted vesicle	2/33	12/19659	1.78E-04	3.17E-03	1.97E-03	DNAJC5/SLC32A1	2
GO:0044391	Ribosomal subunit	4/33	191/19659	2.83E-04	4.47E-03	2.78E-03	RPS3/RPS20/HBA1/HBA2	4
GO:0071682	Endocytic vesicle lumen	2/33	19/19659	4.59E-04	6.52E-03	4.06E-03	HBA1/HBA2	2
GO:0005840	Ribosome	4/33	276/19659	1.13E-03	1.46E-02	9.07E-03	RPS3/RPS20/HBA1/HBA2	4
GO:0060205	Cytoplasmic vesicle lumen	4/33	338/19659	2.37E-03	2.62E-02	1.63E-02	FGA/ALB/HBA1/HBA2	4
GO:0031983	Vesicle lumen	4/33	339/19659	2.40E-03	2.62E-02	1.63E-02	FGA/ALB/HBA1/HBA2	4
GO:0098563	Intrinsic component of synaptic vesicle membrane	2/33	46/19659	2.70E-03	2.74E-02	1.71E-02	DNAJC5/SLC32A1	2
GO:0035577	Azurophil granule membrane	2/33	58/19659	4.26E-03	4.03E-02	2.51E-02	DNAJC5/TMEM30A	2
GO:0030658	Transport vesicle membrane	3/33	204/19659	4.78E-03	4.24E-02	2.64E-02	DNAJC5/SLC32A1/TMEM30A	3
GO:0031093	Platelet alpha granule lumen	2/33	67/19659	5.64E-03	4.71E-02	2.94E-02	FGA/ALB	2
GO:0031300	Intrinsic component of organelle membrane	3/33	226/19659	6.34E-03	4.98E-02	3.10E-02	DNAJC5/SLC32A1/ITM2B	3
GO:0005844	Polysome	2/33	73/19659	6.67E-03	4.98E-02	3.10E-02	VIM/RPS3	2

*GeneRatio = The ratio of the number of genes enriched by the CC to the total number of genes enriched. BgRatio = The ratio of the number of genes contained in the CC to the number of genes in the BP database*.

**Table 4:** Statistically significant GO biological processes (BP) derived from ubiquitinated proteins in human pituitary adenomas.

**ID**	**Biological process**	**GeneRatio**	**BgRatio**	***P-*value**	***P*.adjust**	***Q*-value**	**GeneID**	**Count**
GO:0097237	Cellular response to toxic substance	5/30	235/18493	3.49E-05	1.66E-02	1.27E-02	ALB/RPS3/HBA1/HBA2/PDCD10	5
GO:0042542	Response to hydrogen peroxide	4/30	141/18493	7.61E-05	1.66E-02	1.27E-02	RPS3/HBA1/HBA2/PDCD10	4
GO:0045739	Positive regulation of DNA repair	3/30	61/18493	1.30E-04	1.66E-02	1.27E-02	H2AFX/RPS3/UBE2N	3
GO:0042983	Amyloid precursor protein biosynthetic process	2/30	11/18493	1.39E-04	1.66E-02	1.27E-02	ITM2C/ITM2B	2
GO:0042984	Regulation of amyloid precursor protein biosynthetic process	2/30	11/18493	1.39E-04	1.66E-02	1.27E-02	ITM2C/ITM2B	2
GO:0046677	Response to antibiotic	5/30	323/18493	1.57E-04	1.66E-02	1.27E-02	RPS3/HBA1/PRL/HBA2/PDCD10	5
GO:0010561	Negative regulation of glycoprotein biosynthetic process	2/30	12/18493	1.66E-04	1.66E-02	1.27E-02	ITM2C/ITM2B	2
GO:0031581	Hemidesmosome assembly	2/30	12/18493	1.66E-04	1.66E-02	1.27E-02	LAMC2/PLEC	2
GO:0015671	Oxygen transport	2/30	15/18493	2.64E-04	2.11E-02	1.62E-02	HBA1/HBA2	2
GO:1903019	Negative regulation of glycoprotein metabolic process	2/30	15/18493	2.64E-04	2.11E-02	1.62E-02	ITM2C/ITM2B	2
GO:0015893	Drug transport	4/30	217/18493	3.98E-04	2.56E-02	1.96E-02	HBA1/HBA2/SLC32A1/TMEM30A	4
GO:0015669	Gas transport	2/30	19/18493	4.28E-04	2.56E-02	1.96E-02	HBA1/HBA2	2
GO:2001022	Positive regulation of response to DNA damage stimulus	3/30	92/18493	4.39E-04	2.56E-02	1.96E-02	H2AFX/RPS3/UBE2N	3
GO:0000302	Response to reactive oxygen species	4/30	224/18493	4.48E-04	2.56E-02	1.96E-02	RPS3/HBA1/HBA2/PDCD10	4
GO:0098869	Cellular oxidant detoxification	3/30	101/18493	5.77E-04	3.08E-02	2.36E-02	ALB/HBA1/HBA2	3
GO:1990748	Cellular detoxification	3/30	105/18493	6.46E-04	3.23E-02	2.47E-02	ALB/HBA1/HBA2	3
GO:0006282	Regulation of DNA repair	3/30	115/18493	8.41E-04	3.96E-02	3.03E-02	H2AFX/RPS3/UBE2N	3
GO:0031112	Positive regulation of microtubule polymerization or depolymerization	2/30	29/18493	1.01E-03	4.30E-02	3.30E-02	RPS3/STMN2	2
GO:0098754	Detoxification	3/30	123/18493	1.02E-03	4.30E-02	3.30E-02	ALB/HBA1/HBA2	3
GO:0042744	Hydrogen peroxide catabolic process	2/30	32/18493	1.22E-03	4.77E-02	3.66E-02	HBA1/HBA2	2
GO:0051291	Protein heterooligomerization	3/30	132/18493	1.25E-03	4.77E-02	3.66E-02	IDE/HBA1/HBA2	3

*GeneRatio = The ratio of the number of genes enriched by the BP to the total number of genes enriched. BgRatio = The ratio of the number of genes contained in the BP to the number of genes in the BP database*.

**Table 5:** Statistically significant GO molecular functions (MF) derived from ubiquitinated proteins in human pituitary adenomas.

**ID**	**Molecular functions**	**GeneRatio**	**BgRatio**	***P*-value**	***P*.adjust**	***Q*-value**	**GeneID**	**Count**
GO:0019825	Oxygen binding	3/31	36/17632	3.38E-05	4.97E-03	3.48E-03	ALB/HBA1/HBA2	3
GO:0031720	Haptoglobin binding	2/31	10/17632	1.33E-04	8.10E-03	5.68E-03	HBA1/HBA2	2
GO:0001540	Amyloid-beta binding	3/31	61/17632	1.65E-04	8.10E-03	5.68E-03	IDE/ITM2C/ITM2B	3
GO:0005344	Oxygen carrier activity	2/31	14/17632	2.69E-04	9.87E-03	6.93E-03	HBA1/HBA2	2
GO:0043177	Organic acid binding	4/31	204/17632	4.29E-04	1.12E-02	7.84E-03	ALB/PAM/HBA1/HBA2	4
GO:0016209	Antioxidant activity	3/31	86/17632	4.56E-04	1.12E-02	7.84E-03	ALB/HBA1/HBA2	3
GO:0050699	WW domain binding	2/31	31/17632	1.35E-03	2.83E-02	1.99E-02	NDFIP1/TCEAL2	2
GO:0048037	Cofactor binding	5/31	495/17632	1.59E-03	2.92E-02	2.05E-02	ALB/PAM/RPS3/HBA1/HBA2	5
GO:0140104	Molecular carrier activity	2/31	43/17632	2.58E-03	4.22E-02	2.96E-02	HBA1/HBA2	2

*GeneRatio = The ratio of the number of genes enriched by the MF to the total number of genes enriched. BgRatio = The ratio of the number of genes contained in the MF to the number of genes in the BP database*.

### Characterization of Ubiquitinated Peptides

**Figure 7:**
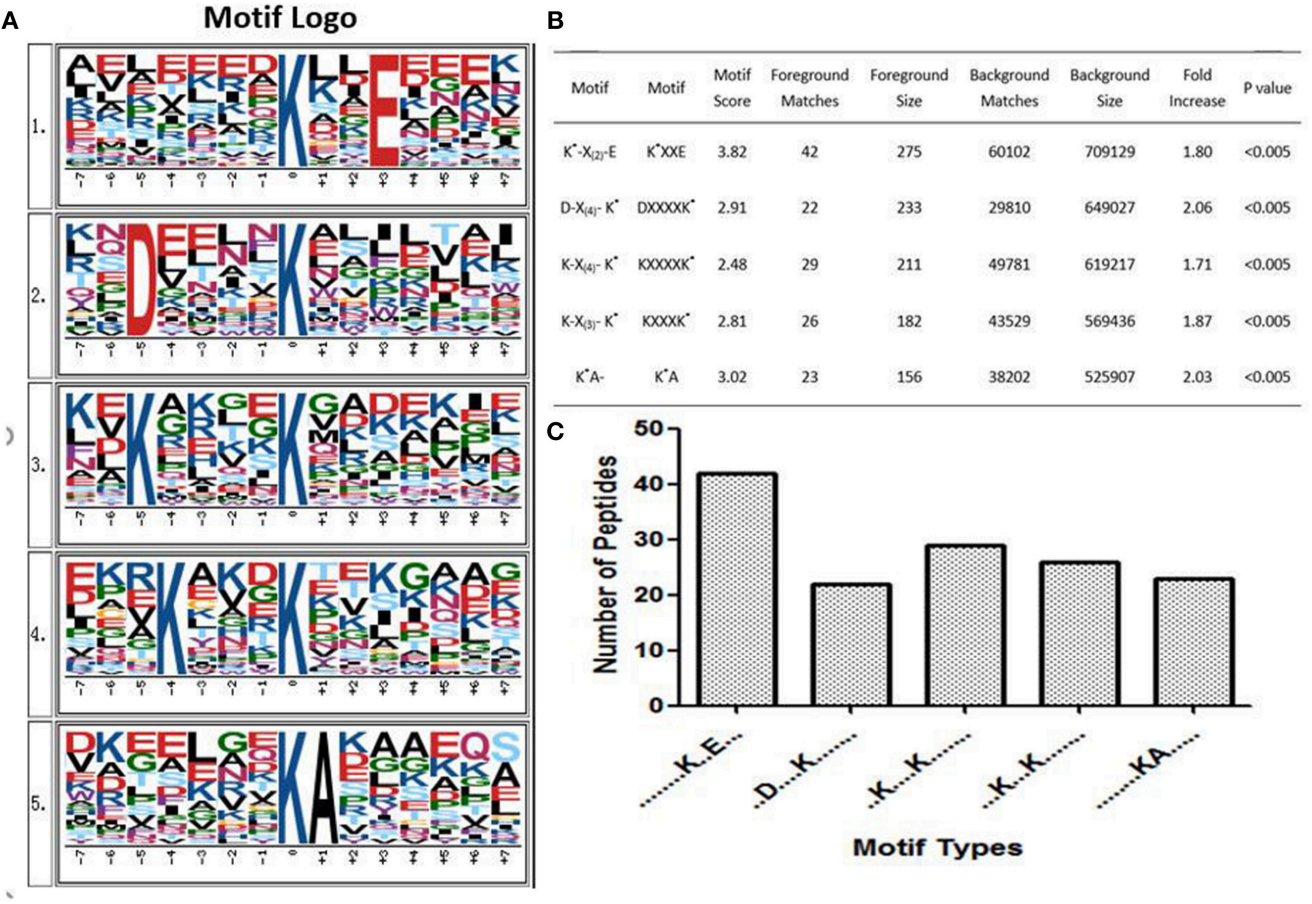
Ubiquitinated protein motifs in human. **(A)** Ubiquitination motifs and the conservation of ubiquitination sites. The central K stands for the ubiquitinated protein. The size of each letter is related to the frequency of amino acid residues occurring at that position. **(B)** Taking the ubiquitinated peptide sequence as a foreground, the sequence window of the ubiquitinated related protein non-ubiquitinated lysine was used as a background control. **(C)** The number of identified ubiquitinated peptides in each motif. K* = ubiquitinated lysine residue. X = any amino acid residue.

Some studies showed that conservative ubiquitination motifs might not exist in humans (34, 40, 54). To elucidate regulation of ubiquitination in human PAs, ubiquitination motif analysis was carried out by examining the sequences from −15 to +15 amino acid residues in the ubiquitination sites of the 142 ubiquitinated peptides with Motif-X software. Five significantly distinguished motifs were identified (Figures 7A,B), including K^*^-X_(2)_-E, D-X_(4)_-K^*^, K-X_(4)_-K^*^, K-X_(3)_-K^*^, and K^*^A, which refers to 42, 22, 29, 26, and 23 unique ubiquitinated peptides, respectively (K^*^ = the ubiquitinated lysine residue; X = any amino acid residue). Those ubiquitinated peptides had different abundances, which together accounted for 99.3% of the identified ubiquitinated peptides (Figure 7C). Although Zhang et al. studied the ubiquitination modification of wheat (55), the ubiquitination motifs of wheat are completely different from the human ubiquitination motif. This result might reveal differences in ubiquitination motifs among different species. The ubiquitination motifs of human proteins obtained in this study might provide ubiquitin-binding loci for future research. However, one must realize that because this study incorporated a small number of ubiquitinated peptides, the characterized human protein ubiquitination motifs still need to be validated from a large number of ubiquitinated peptide sequences.

### Further Analysis of Ubiquitinated Proteins in Pituitary Adenomas

**Figure 8:**
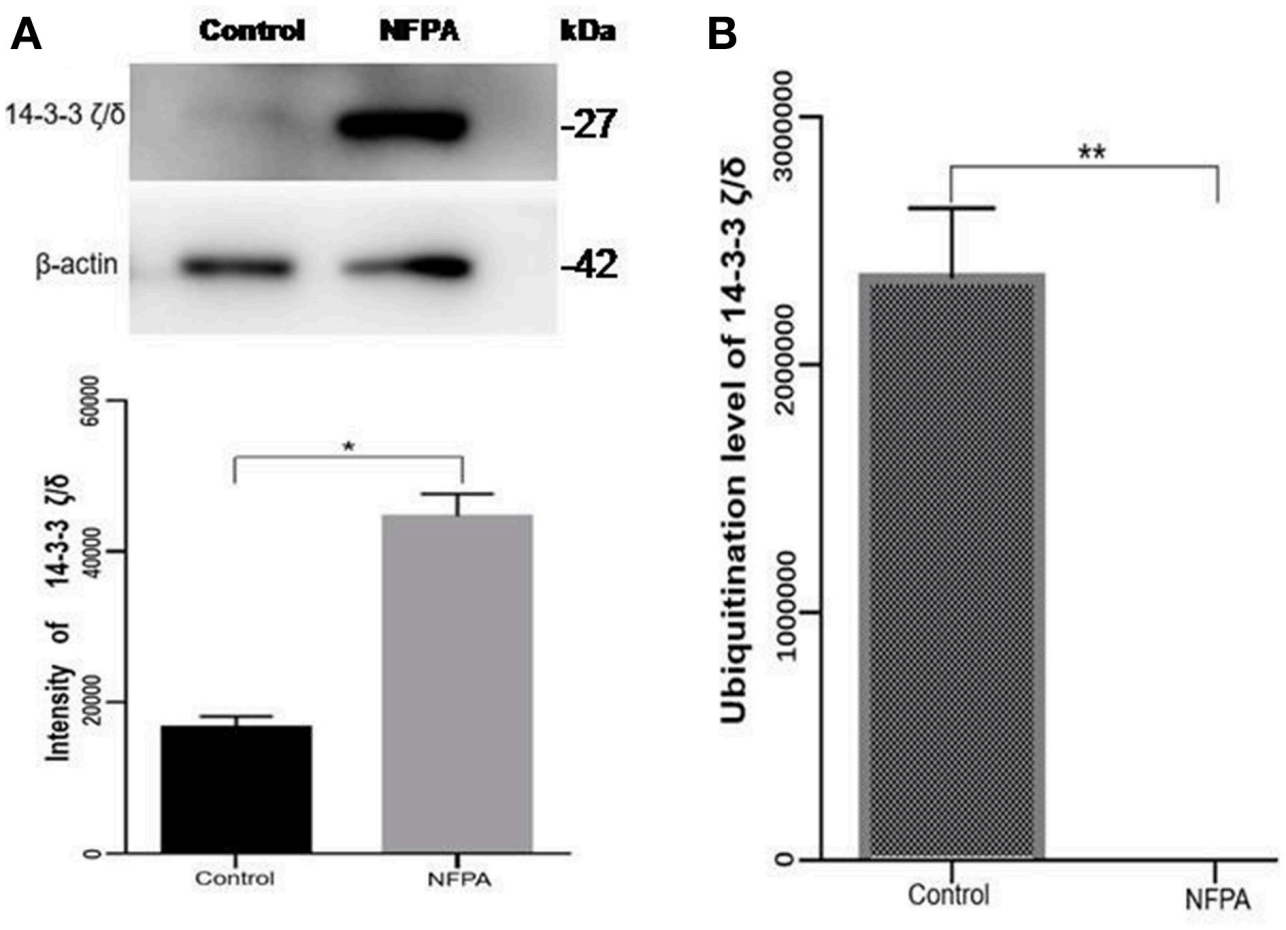
The protein expression level and its ubiquitinated level of protein 14-3-3 zeta/delta in NFPAs compared to controls. **(A)** Western blot analysis of the protein expression level of protein 14-3-3 zeta/delta in NFPAs compared to controls. **(B)** The ubiquitination level of protein 14-3-3 zeta/delta in NFPAs compared to controls. *p < 0.05. **p < 0.01. NFPA, nonfunctional pituitary adenomas; Control, control pituitaries.

After comprehensive analysis of ubiquitination data, KEGG pathways, and GO enrichment data, eight statistically significant KEGG signaling pathways (*p* < 0.05) were identified. Only four of these enriched signaling pathways were associated with tumors, and the protein 14-3-3 zeta/delta was an important molecule in the PI3K-AKT signaling pathway and the Hippo signaling pathway. Also, the peptide from protein 14-3-3 zeta/delta underwent ubiquitination in control pituitary tissues, but not in NFPA tissues (Table 2). Therefore, the ubiquitinated protein 14-3-3 zeta/delta was chosen for further analysis with Western immunoaffinity blot. The result showed that protein 14-3-3 zeta/delta was significantly upregulated in NFPAs compared to controls. Quantitative ubiquitinated proteomics showed that the peptide from protein 14-3-3 zeta/delta was ubiquitinated in controls but not in NFPAs (Figure 8; Table 2). The decreased ubiquitination level of protein 14-3-3 zeta/delta in NFPAs might inhibit degradation of this protein and change the signaling transduction of this protein in PAs.

## Discussion

### The Functions of Protein Ubiquitination

Ubiquitination in a protein is under a wide range of functions, and regulates a variety of basic cellular processes, including gene transcription, DNA repair and replication, protein degradation, viral particle sprouting, and intracellular trafficking (56). Monoubiquitination is involved in the regulation of lysosome targeting, endocytosis, and chromatin remodeling and meiosis. Polyubiquitination involves DNA damage and repair, targets modified proteins to proteasomal degradation, and includes immune signal transduction (17). Chen et al. suggest that ubiquitination has become a key regulator of the immune system involved in transduction of intracellular signals, control of T cell differentiation, and induction of immune tolerance (57). The ubiquitination regulatory pattern recognizes receptor signaling, initiates adaptive immune responses, and maturates dendritic cells required to mediate innate immune responses. For T cells, ubiquitination regulates their development, activation, and differentiation to thereby maintain immune tolerance to their own tissues and an effective adaptive immune response to pathogens (20). The role of ubiquitination in immune regulation was first discovered in studies of antigen presentation and transcription factor nuclear factor NFκB family (58). The transcription factor nuclear factor NF-κB controls basic functions of many cells, including cell proliferation, immune responses, and apoptosis (59). Excessive apoptosis can result in anemia, neurodegenerative diseases, and graft rejection. A reduction of apoptosis can lead to autoimmune diseases and cancer. Therefore, moderate apoptosis is of great importance to the body. Ubiquitination of apoptotic proteins is a key component of the apoptosis signaling cascade (60). The ubiquitination mentioned above has an effect on DNA damage and repair. Improper response of DNA damage might accelerate the aging process, cause genomic instability, and eventually lead to various human diseases, including neurodegenerative diseases and cancer (61). Ubiquitination plays an important role to regulate the tumor suppressor function of Beclin1 (62). Thus, ubiquitination might play a crucial part in cancer. Some publications have described that ubiquitination disorders affect the occurrence, development, and metastasis of cancer (22, 63, 64).

### Ubiquitinated Proteins Regulated Diverse Biological Process

This study found the mainly GO biological processes were related to synthesis and metabolism of proteins, which included glycoprotein, amyloid precursor protein, regulation of proteasomal ubiquitin-dependent protein, etc. This result suggests that ubiquitinated proteins might be involved in the synthesis and metabolism of certain proteins. Our long-term proteomics studies found that the number of down-regulated proteins was much more than up-regulated proteins in different subtypes of NFPAs compared to control pituitaries (28), mRNA expressions of ubiquitin-conjugating enzymes E2 and E3 were significantly increased in NFPAs (28), mRNA expression of ubiquitin specific protease 34 was significantly decreased in PAs (29), proteasome subunit alpha type 2 was nitrated in PAs (26), and the protein ubiquitination pathway was changed in NFPAs (30). It is well-known that synthesis and degradation of proteins in humans maintain in a dynamic balance. The increased number of down-regulated proteins in PAs might mean a disrupted balance between synthesis and degradation of proteins compared to control pituitaries. This study clearly found that ubiquitinated proteins in PAs were related to the synthesis and metabolism of proteins. The ubiquitinated proteasome system is one of the main pathways for intracellular protein degradation (8, 65, 66). Ubiquitination can achieve protein degradation by ubiquitinating the proteasome. Therefore, the increased number of these downregulated proteins in human NFPAs might undergo ubiquitination to result in more degradation of the proteins relative to normal pituitaries.

The phosphatidylinositol 3-kinase (PI3K)-AKT signaling pathway is activated by many types of cellular and toxic damage and regulates basic cellular functions such as transcription, translation, proliferation, growth, and survival. Binding of growth factors to their receptor tyrosine kinase or G protein-coupled receptor stimulates the Ia and Ib PI3K subtypes, respectively. PI3K catalyzes the production of phosphatidylinositol-3, 4, 5-triphosphate on the cell membrane. PIP3 in turn acts as a second messenger to activate AKT. The activated AKT can control key cellular processes through phosphorylation involved in apoptosis, protein synthesis, metabolism, and cell cycle substrates. The PI3K-AKT signaling pathway is an important signaling pathway in cells, and its main function is to inhibit apoptosis and promote proliferation. In various malignant tumors, the PI3K-AKT signaling pathway is abnormally regulated to promote formation of new blood vessels, proliferation of tumor cells, and inhibition of apoptosis, and is closely related to tumor metastasis and invasion. The HVP90 inhibitor NVPAUY922 and the PI3K-mTOR inhibitor NVP-BEZ235, alone or in combination, have a significant effect on the apoptosis of cholangiocarcinoma cells; and these inhibitors act on the rat model of cholangiocarcinoma to decrease the tumor (67). These results indirectly indicate that activation of PI3K-AKT signaling pathway contributes to the proliferation of cholangiocarcinoma cells. Study also found that the P110α subunit of PI3K is a regulator of angiogenesis, and the inactivation of P110α leads to non-functional angiogenesis, which in turn prevents tumor growth (68). In the PI3K-AKT signaling pathway, the key molecules are cytokine, ECM, ITGB, and 14-3-3. Lamnin subunit gamma2 (LAMC2), which is a molecule in ECM, has been reported to be involved in the development and progression of various tumors (69). Smith et al. (70) found that LAMC2 was associated with bladder cancer metastasis, and its expression level increased with an increase of human tumor stage. In colorectal cancer, stable overexpressed LAMC2 promotes proliferation, migration, and invasion of cancer cells (71). The grade of LAMC2 expression was significantly associated with the pattern and depth of invasion of oral squamous cell carcinoma (72). This study found that LAMC2 was ubiquitinated at position 219. PAs are generally benign tumors, and do not metastasize; however, they do proliferate and invade of tumor cells.

The core components of the Hippo signaling pathway include upstream and downstream regulatory factors, core kinase cassettes, and downstream oncogenes. The core kinase cassette includes Lats1/2, Mst2, SAV1, and Mob. Activation of the Lats1/2 phosphorylation of the transcriptional coactivators YAP and TAZ ultimately leads to apoptosis, limits organ size overgrowth, or promotes cell growth and proliferation (73). Therefore, the Hippo signaling pathway prevents tissue growth and tumorigenesis (74). However, the abnormality of this pathway usually leads to the occurrence of tumors (75). For example, when Lats1 is ubiquitinated, the kinase activity of Lats1 is reduced and subsequently inhibited by Hippo signaling only promotes cell proliferation, but also inhibits cell apoptosis and attenuates tumor suppressor function (76). NEDD4, an E3 ubiquitin ligase, can directly interact with Lats1 to lead to its ubiquitination and decreased levels of Lats to thereby increase the localization of nuclear YAP, and activate proliferative and anti-apoptotic genes (77). Lignitto et al. (78) found that the ubiquitination-proteasome system can degrade Mob to attenuate the Hippo cascade and maintain the growth of glioblastoma cells *in vivo*. So what is the impact of ubiquitination on the Hippo signaling pathway? Toloczko et al. (73) found that USP9X, a deubiquitinating protease, can enhance LATS kinase to inhibit tumor growth. Therefore, the Hippo signaling pathway is closely related to tumorigenesis, and the ubiquitination and deubiquitination of the core kinase cassette in the Hippo signaling pathway have a great influence on tumor growth. Therefore, ubiquitination and deubiquitination of the core kinase cassette are worthy of further study, and might lead to the development of new treatments for tumors. In addition, some key molecules in the Hippo signaling pathway are 14-3-3 protein and F-actin. The 14-3-3 protein in the Hippo signaling pathway is closely related to tumors.

### The Ubiquitination of 14-3-3 Proteins in Pituitary Adenomas

Humans 14-3-3 proteins have many subtypes, including 14-3-3 protein beta/alpha, 14-3-3 protein gamma, 14-3-3 protein theta, etc. 14-3-3 subtypes are considered to play nocogenic roles in a variety of tumors (79). Raungrut et al. (80) found that 14-3-3 gamma is involved in the metastasis of lung cancer cells, and found that knockdown of 14-3-3 gamma could inhibit lung cancer metastasis. The 14-3-3 beta protein has been shown to possess carcinogenic potential, and its increased expression has been detected in many types of cancers. Tang et al. (81) found that 14-3-3 beta promotes migration and invasion of human hepatocellular carcinoma cells by modulating expression of MMP2 and MMP9 through the PI3K/Akt/NF-κB pathway. Also, 14-3-3 τ can promote breast cancer invasion and metastasis by inhibiting RhoGDI (82). However, few studies are involved in the relationship of 14-3-3 zeta/delta proteins and tumorigenesis. This study found the 14-3-3 zeta/delta protein was ubiquitinated in pituitary control tissues but not in PA tissues. However, Western blot analysis found that 14-3-3 zeta/delta protein was highly expressed in NFPAs compared to control tissues. The ubiquitinated proteasome system is one of the major pathways for intracellular protein degradation (8, 65, 66). Ubiquitination can achieve protein degradation by ubiquitination of the proteasome. Thus, it is hypothesized that proteins can be degraded by ubiquitination modification to result in lower protein levels in tumors than control tissues. Therefore, up-regulated expression of 14-3-3 zeta/delta protein in NFPAs might be due to the decreased ubiquitination level, and contribute to pituitary tumorigenesis. These findings might provide a better basis for biomarker discovery and the early treatment of PA patients.

### Strengths and Limitations of This Study

This study, for the first time, used anti-ubiquitin antibody (specific to K-ε-GG)-based label-free quantitative proteomics to identify protein ubiquitination profiling between NFPAs and control pituitaries. A total of 158 ubiquitinated sites in 108 ubiquitinated proteins was identified and quantified, which is the first ubiquitinome profile in NFPAs compared to controls. Further, pathway network analysis revealed alterations of multiple ubiquitination-involved signaling pathway systems in NFPAs to offer novel insights into molecular mechanisms of NFPAs and to provide a new source to discover new biomarkers for NFPAs. However, one must realize that a ubiquitinome is dynamic, and varies with different conditions and pathophysiological status. PAs are highly heterogeneous among tumor individuals, different subtype of NFPAs, and different subtypes of FPAs. In order to further in-depth insight into functional significance of each ubiquitination in PA pathogenesis, one must significantly expand the number of human tissue samples studied to validate and quantify each ubiquitination among individuals and different PA subtypes; also, biological functions of each ubiquitination should be examined in the cell model and animal model. For this current study, due to the very limited, precious pituitary adenoma and control tissue samples, only very limited amount of proteins were used for trypsin digestion, anti-ubiquitin antibody-based enrichment, and LC-MS/MS analysis. The number of ubiquitinated sites and ubiquitinated proteins might be significantly increased with an increased amount of proteins in future ubiquitinomics analysis among PA individuals and among different PA subtypes, to significantly expand the ubiquitinome database of PAs, which will offer the increased opportunity to in-depth explore biological roles of protein ubiquitination in PAs.

## Conclusion

Ant-ubiquitin antibody-based label-free quantitative proteomics effectively identified and quantified protein lysine ubiquitination in human PAs compared to controls. This study provides the first protein ubiquitination profiling of human PAs and control pituitaries to understand ubiquitination-mediated multiple cellular functions and biological processes. This study expanded the range of physiological processes regulated by ubiquitination, and serves as a valuable reference for biological functions of protein ubiquitination in human PAs.

## Ethics Statement

PA tissues were obtained from the Department of Neurosurgery of Xiangya Hospital, China, as approved by the Xiangya Hospital Medical Ethics Committee of Central South University. Control pituitary tissues were obtained from the Memphis Regional Medical Center (*n* = 5), as approved by University of Tennessee Health Science Center Internal Review Board. The consent was attained from each patient or the family of each control pituitary subject (post-mortem tissues) after full explanation of the purpose and nature of all experimental procedures.

## Author Contributions

SQ analyzed data, carried out Western blot experiment, prepared figures and tables, designed and wrote the manuscript. XhZ participated in the analysis, and revised the manuscript. ML and NL participated in partial data analysis. YL prepared the protein samples. XL collected tissue samples and performed clinical diagnosis. DD provided the control tissues, and critically evaluated and revised the manuscript. XqZ conceived the concept, designed experiments and manuscript, instructed experiments, analyzed data, obtained the ubiquitinated proteomic data, supervised results, coordinated, wrote and critically revised manuscript, and was responsible for its financial supports and the corresponding works. All authors approved the final manuscript.

### Conflict of Interest Statement

The authors declare that the research was conducted in the absence of any commercial or financial relationships that could be construed as a potential conflict of interest.

## Abbreviations

BP: biological process
CC: cellular component
DTT: dithiothreitol
FPA: functional pituitary adenoma
GO: gene ontology
HCD: higher-energy collisional dissociation
HPLC: high performance liquid chromatography
IAP: immunoaffinity purification
LAMC2: lamnin subunit gamma 2
LC: liquid chromatography
MF: molecular function
MS/MS: tandem mass spectrometry
NER: nucleotide excision repair
NFPA: nonfunctional pituitary adenoma
PA: pituitary adenoma
PI3K: phosphatidylinostiol 3-kinase
PTMs: post-translational modifications
TFA: trifluoroacetate.
